# Expression of a recombinant endolysin from bacteriophage CAP 10-3 with lytic activity against *Cutibacterium acnes*

**DOI:** 10.1038/s41598-023-43559-z

**Published:** 2023-09-30

**Authors:** Ja-I Kim, Muhammad Adeel Hasnain, Gi-Seong Moon

**Affiliations:** 1 Major of Biotechnology, Korea National University of Transportation, Jeungpyeong, 27909 Korea; 2 Major in IT·Biohealth Convergence, Department of IT·Energy Convergence, Graduate School, Korea National University of Transportation, Chungju, 27469 Korea

**Keywords:** Genetics, Microbiology

## Abstract

The bacteriophage CAP 10-3 forming plaques against *Cutibacterium acnes* which causes skin acne was previously isolated from human skin acne lesion. Incomplete whole genome sequence (WGS) of the bacteriophage CAP 10-3 was obtained and it had 29,643 bp long nucleotide with 53.86% GC content. The sequence was similar to *C. acnes* phage PAP 1-1 with a nucleotide sequence identity of 89.63% and the bacteriophage belonged to *Pahexavirus*. Bioinformatic analysis of the WGS predicted 147 ORFs and functions of 40 CDSs were identified. The predicted endolysin gene of bacteriophage CAP 10-3 was 858 bp long which was deduced as 285 amino acids (~ 31 kDa). The protein had the highest similarity with amino acid sequence of the endolysin from *Propionibacterium* phage PHL071N05 with 97.20% identity. The CAP 10-3 endolysin gene was amplified by PCR with primer pairs based on the gene sequence, cloned into an expression vector pET-15b and transformed into *Escherichia coli* BL21(DE3) strain. The predicted protein band (~ 33 kDa) for the recombinant endolysin was detected in an SDS-PAGE gel and western blot assay. The concentrated supernatant of cell lysate from *E. coli* BL21(DE3) (pET-15b_CAP10-3 end) and a partially purified recombinant CAP 10-3 endolysin showed antibacterial activity against *C. acnes* KCTC 3314 in a dose-dependent manner. In conclusion, the recombinant CAP 10-3 endolysin was successfully produced in *E. coli* strain and it can be considered as a therapeutic agent candidate for treatment of human skin acne.

## Introduction

*Cutibacterium acnes* is a Gram-positive aerotolerant anaerobic bacterium. *C. acnes* is mainly found in the areas of the skin rich in sebaceous glands; it accounts for about 90% of the total skin microbiota making it the most abundant bacterium^1,2^. Its involvement in several disorders like eye infections, prostate cancer, acne vulgaris^3^ sarcoidosis^4^ and rarely bacterial endocarditis^5^ has caused its status shifted from commensal bacterium to an opportunistic pathogen^6^. Acne vulgaris is the most abundant skin disorder mainly effecting adolescents. Besides being a skin disorder, it can prove to be an emotional, psychological and social challenge causing depression^7,8^. *C. acnes* is believed to be the main contributor in the pathology of acne vulgaris where it leads to the formation of acne lesions by eliciting inflammatory response. It’s conventional treatment with topical and systematic antibiotic administration has led to an upsurge in resistance and detection of antibiotic resistant *C. acnes* strains has become increasingly common^9–12^ necessitating search of new non-conventional alternatives among which, phage therapy can be a promising option.

Although phage therapy as a potential antibacterial treatment has been around since pre-antibiotic era (since almost a decade before the first antibiotic was discovered), discovery and widespread successful use of antibiotics caused phages’ therapeutic use limited to the Soviet Union^13^. For many years, poorly understood phage biology, lack of quality control and inconsistent and unreliable trials caused the phage therapy to lose attention and phage use was predominantly limited for the research purpose^14^.

However, ever-rising problem of antibiotic resistance has made them a potential candidate to replace or augment the antibiotic treatment^15^. Renewed research interest in the phage therapy in last two decades has come up with its several promising advantages that include host specificity, exponential rate of replication, reproducibility^16^, quick elimination from the body in the absence of host^17^, reduced toxicity to humans, ability to destroy biofilms^18^ and disrupt vegetative cells^19^. Recent research suggests that phages can be used to increase the efficacy of the antibiotics^16^ and in some cases revert the antimicrobial resistance^20^.

Since using the whole phage poses numerous challenges yet to be addressed like immune response, inactivation by sterilization etc., one possible alternative is to use phage lytic enzymes only^14^. Endolysin is a phage protein capable of lysing target bacteria and has received a considerable attention as a potential antimicrobial agent. One of their recently discovered capability is to cure hard-to-treat infections by crossing cell membranes of epithelial cells^21^.

Since 2001, a number of successful pre-clinical trials have been conducted using lysins to inhibit mucosal colonization of pathogenic bacteria which shows their potential to at-least complement the conventional antibiotic treatment strategies^22–26^. In the current study, whole genome sequencing of previously isolated *C. acnes* phage CAP 10-3 was carried out followed by its characterization. A total of 147 ORFs were predicted, most of whose proteins showed homology with the already characterized *C. acnes* bacteriophage PAP 1-1 (GenBank accession no., OP491959). The predicted endolysin gene was successfully amplified, cloned and expressed in *E. coli*. The recombinant protein demonstrated effective lytic activity against *C. acnes* KCTC 3314 strain.

## Materials and methods

### Bacterial strains and culture conditions

The bacteriophage CAP 10-3 forming plaques against *C. acnes* was previously isolated from human skin acne lesions. *C. acnes* KCTC 3314 strain which was purchased from the Korean Collection for Type Cultures (KCTC) was used in this study. The strain was cultured in Reinforced Clostridial Medium (RCM) (BD, Sparks, MD, USA) in an anaerobic chamber (DG250; Don Whitley Scientific Ltd., Bingley, UK). *E. coli* BL21 (DE3) was purchased from Enzynomics Co. Ltd. (Daejeon, Korea) and used for protein expression. The strain was grown in Luria–Bertani (LB) broth (BD) and incubated at 37 °C.

### Genomic characterization of bacteriophage CAP 10-3

A phage DNA isolation kit (Norgen Biotek Co., Thorold, Canada) was used to isolate phage CAP 10-3 genomic DNA. The isolated genomic DNA was used for whole genome sequencing on iSeq 100 sequencer (Illumina, Inc., San Diego, CA, USA). A library was created for sequencing and the sequences were assembled using the local run manager assembly program (Ver. 1.0.0). The library was prepared using an Illumina prep kit using BLT (bead-linked transposomes) method according to the manufacturer’s guide (Illumina). Briefly, BLT was used to fragment and tag the DNA with adapter sequence followed by the post tagmentation clean-up using TSB (tagment stop buffer) and TWB (tagment wash buffer). Tagmented DNA was then amplified using limited-cycle PCR (5 cycles) where index adapters and sequences required to generate sequencing cluster were also added. The prepared library was subsequently purified using SPB (sample purification beads) and used for sequencing. Subsequently, protein coding sequences (CDS) were identified by using PHASTER (https://phaster.ca). NCBI BLAST (https://blast.ncbi.nlm.nih.gov/Blast.cgi) and Uniprot (https://www.uniprot.org) were used to confirm gene homology and function. Molecular weight and isoelectric point of a putative endolysin were confirmed using ExPASy (https://www.expasy.org). The whole genome sequence of bacteriophage CAP 10-3 was deposited to the GenBank (accession no., OR039357).

### Construction of *E. coli* expression vector

The genomic DNA of CAP 10-3 was used as the template for PCR amplification of the endolysin gene. The primers CAP10-3_endolysin_1F(A)_NdeI (5ʹ-GGTGGTCATATGAGGTTTATTCC-3ʹ; NdeI recognition sequence is underlined) and CAP10-3_endolysin_R (5ʹ-TCACTTTTTCAAACCGTTGAC-3ʹ) were designed and used for the amplification with a PCR premix kit (i-pfu, Intron Biotechnology, Inc., Seongnam, Korea). PCR running conditions were as follows: 95 °C, 5 min for pre-denaturation; 30 cycles of 95 °C-30 s, 55 °C-30 s, and 72 °C-1 min; 72 °C, 5 min for final extension. The PCR products digested with NdeI restriction endonuclease (Beams Biotechnology Co., Ltd., Seongnam, Korea) were ligated with pET-15b expression vector (Novagen, Massachusetts, USA) which was sequentially treated with BamHI restriction endonuclease (Beams Biotechnology), Klenow fragment (New England Biolabs, Boston, MA, USA), and NdeI endonuclease (Beams Biotechnology) by T4 DNA ligase (Bioneer Co., Daejeon, Korea). The recombinant DNA was introduced to the competent *E. coli* BL21(DE3) by heat shock method according to the manufacturer’s instructions. Transformants were selected on LB agar plate supplemented with 100 μg/mL of ampicillin and confirmed by PCR and restriction profile.

### Expression of recombinant endolysin in *E. coli*

*E. coli* BL21(DE3) transformed with pET-15b_CAP10-3 end for expression of the endolysin gene of bacteriophage CAP 10-3 was inoculated in 100 mL of LB broth containing 100 μg/mL of ampicillin and incubated at 37 °C with 250 rpm to 0.6 of OD_600._ For induction of endolysin gene expression, 1 mM of isopropyl-β-d-thiogalactopyranoside (IPTG) was added to the culture and incubated at 37 °C with 150 rpm for 4 h (a control culture without the induction was also prepared). The cultures were centrifuged at 5000*g* for 7 min and the harvested cell pellets and cell lysates were analyzed by SDS-PAGE and Western blot assay to confirm whether the recombinant endolysin was properly expressed. Briefly, the cell pellet was diluted with 6 × Laemmli sample buffer, an appropriate volume of the suspension was boiled at 95 °C, and the sample was subjected to SDS-PAGE under the condition of 110 V, 80 min. The cell pellet was also suspended in 4 mL of Lysis buffer (50 mM NaH_2_PO_4_, 300 mM NaCl, 10 mM imidazole, pH 8.0) and disrupted on ice with a sonicator (Qsonica, Newtown, CT, USA) by 60% amplitude of 6 cycles of 10 s pulse and 10 s interval. The sonicated sample was centrifuged at 10,000*g* for 20 min at 4 °C, the supernatant was filtered with a syringe filter (0.45 μm; Anylab, Seoul, Korea), and the filtrate was concentrated by Amicon® Ultra-15 Centrifugal Filter Unit (MWCO. 10 K; Millipore, Massachusetts, USA). The concentrate was analyzed by Western blot assay. For the assay, proteins on the SDS-PAGE gel were transferred to Immobilon®-P PVDF membrane (0.45 μm; Merck, Middlesex, USA) and 6 × His-tag recombinant rabbit monoclonal antibody (Invitrogen, Middlesex, USA) and goat anti-rabbit lgG (H + L) secondary antibody with HRP (Invitrogen) were used for detection of His-tagged endolysin in the membrane.

### Purification of recombinant endolysin

His-tagged recombinant endolysin was purified by using Ni–NTA agarose (QIAGEN, Hilden, Germany) according to the manufacturer’s instruction. One milliliter of Ni–NTA slurry was transferred to a 15 mL tube, centrifuged at 2500*g* for 5 min, and the supernatant was removed. Two milliliters of lysis buffer was added to the Ni–NTA resin, inverted 10 times, centrifuged, and the supernatant was removed. The resin was then mixed with 4 mL of cell lysate containing recombinant endolysin. The mixture was reacted by shaking at 4 °C for 1 h and transferred to a column (Bio-Rad) with a bottom cap and the cap was removed to collect the flow-through. The resin was washed twice with 4 mL wash buffer (50 mM NaH_2_PO_4_, 300 mM NaCl, 20 mM imidazole, pH 8.0) and eluted 4 times with 0.5 mL elution buffer (50 mM NaH_2_PO_4_, 300 mM NaCl, 250 mM imidazole, pH 8.0). The eluent was concentrated by using an Amicon® Ultra-15 Centrifugal Filter Unit (MWCO. 10 K; Millipore) and protein quantification was done by BCA protein assay kit (Thermo Fisher Scientific, Massachusetts, USA).

### Evaluation of antibacterial activity of recombinant endolysin

The antibacterial activity of the recombinant endolysin against *C. acnes* KCTC 3314 strain was evaluated by using the turbidity reduction method with minor modification of the original method by Yoong et al.^27^. The mid-log phase of *C. acnes* KCTC 3314 strain (1.8 mL) was centrifuged at 10,000*g* for 5 min and the harvested cell pellet was rapidly frozen in liquid nitrogen and stored at − 20 °C until use or used immediately by suspending in 1.3 mL of 1 × PBS on ice. The cell suspension (270 μL) and 30 μL of the recombinant *E. coli* cell lysate or purified recombinant endolysin were incubated at 37 °C for 2 h. At every 10 min, optical densities at 600 nm were measured by a microplate reader (Synergy HTX Multi-Mode Reader, BioTek, Vermont, USA). Additionally the viable cell counts were measured by tenfold dilution method at times (0, 2, 4, 6, 8 h) in a case.

### Statistical analysis

The experiments in this study were repeated 3 times and expressed as mean ± standard deviation (SD) and statistical analyses were done by using SPSS ver. 25 (Statistical Package for Social Sciences, SPSS Inc., Chicago, IL, USA). The significance was verified by one-way ANOVA, and Duncan's multiple range test (p < 0.05) for absorbance and Scheffe test (p < 0.05) for viable cell count were used for post-hoc.

### Ethical approval

The study was performed in agreement with the Declaration of Helsinki principles and approved by the Korea National University of Transportation-Institutional Review Board (KNUT-IRB2020-19).

## Results

### Bacteriophage CAP 10-3 whole genome sequence

**Figure 1:**
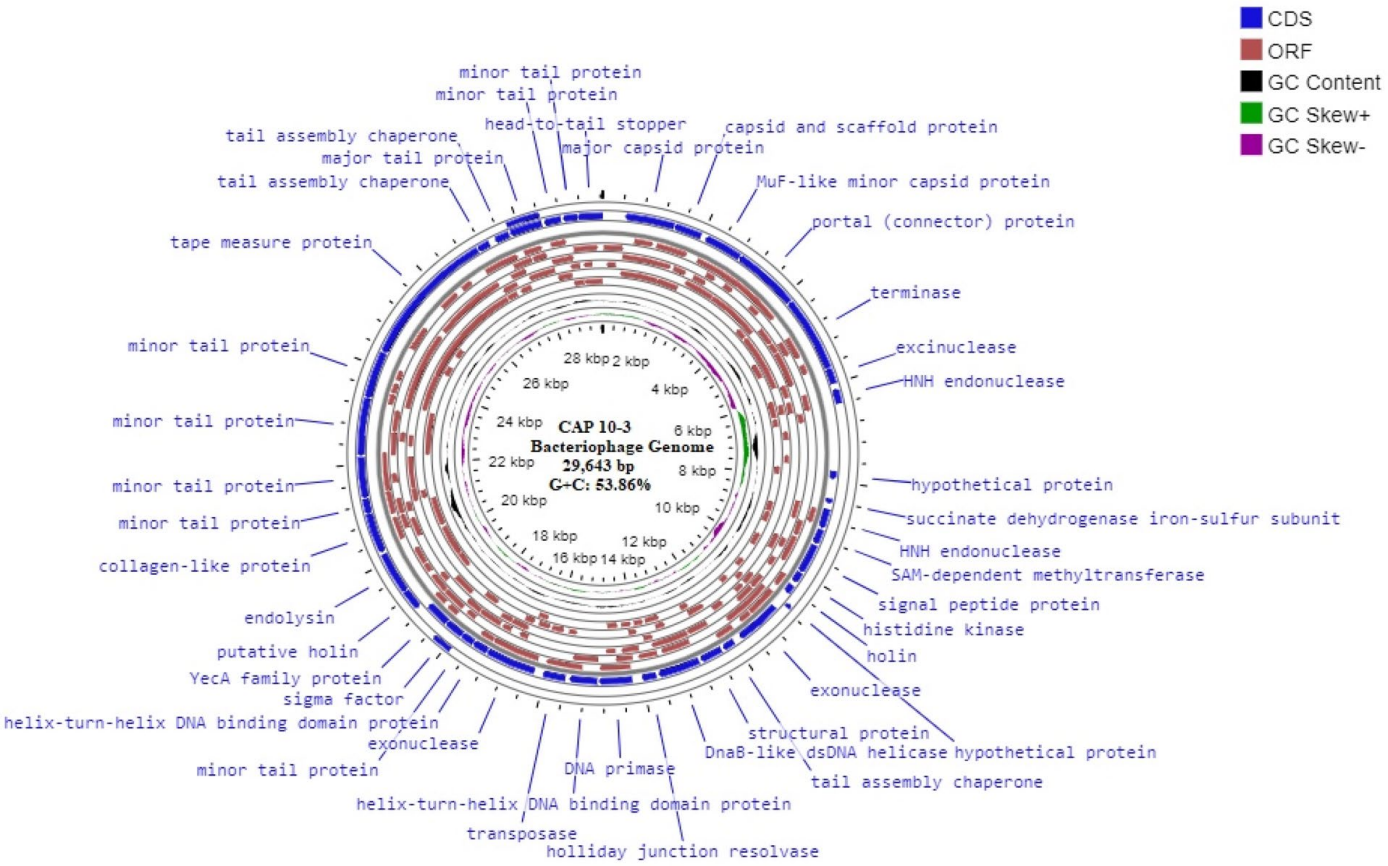
The physical map of whole genome sequence of CAP 10-3 bacteriophage.

The genome of CAP 10-3 consisted of dsDNA of 29,643 bp with a GC content of 53.86% and 89.63% nucleotide sequence similarity to *C. acnes* phage PAP 1-1 (GenBank accession no., OP491959), a species of *Pahexavirus* recently characterized by our group^28^. Homology search using BLASTn showed that it has high degree of similarity to many other *C. acnes* phages as well. A total of 147 ORFs (open reading frames) were predicted, while the functions of 40 out of 42 coding sequences (CDSs) were identified using PHASTER (Fig. 1). Putative holin and endolysin of CAP 10-3 were very similar to those of *C. acnes* phage PAP 1-1 with more than 90% amino acid sequence identity. In addition, other proteins like transcriptional regulators, primase, portal protein, exonuclease, capsid, scaffold protein, and sigma factor also showed a wide range of sequence similarity with those of the phage PAP 1-1. TMP (Tape measure protein), in particular, showed a high degree of homology with those of the previously characterized *C. acnes* phages. The predicted molecular weight of CAP 10-3 endolysin was approximately 31 kDa and the protein had the highest similarity with amino acid sequences of endolysins from *Propionibacterium* phage PHL071N05 (GenBank accession no., YP_008531700.1) and *Cutibacterium* phage P108C (GenBank accession no., QHB37111.1) with 97.20% and 95.45% identity, respectively. It also showed similarities with those of N-acetylmuramoyl-L-alanine amidase from *Caudoviricetes* sp. (GenBank accession no., DAW17174.1) and peptidoglycan recognition family protein from *C. acnes* (GenBank accession no., WP_303622234.1) with 95.80% and 94.41% identity, respectively.

### Construction of recombinant *Escherichia coli* for expression of endolysin

The putative endolysin gene which was amplified by PCR with primers CAP10-3_endolysin_1F(A)_NdeI and CAP10-3_endolysin_R was confirmed by the size (867 bp) on an electrophoresis gel (data not shown). The sequence includes NdeI restriction endonuclease recognition sequence on 5ʹ region of CAP10-3_endolysin_1F(A)_NdeI primer and encodes 285 amino acids. In particular, the start codon (GTG) of original sequence was changed into ATG which is more efficient for start of translation in *E. coli* strains^29^. The recombinant DNA pET-15b_CAP10-3 end was successfully transformed to *E. coli* BL21(DE3) and it resulted in *E. coli* BL21(DE3) (pET-15b_CAP 10-3 end).

### Expression of recombinant endolysin

**Figure 2:**
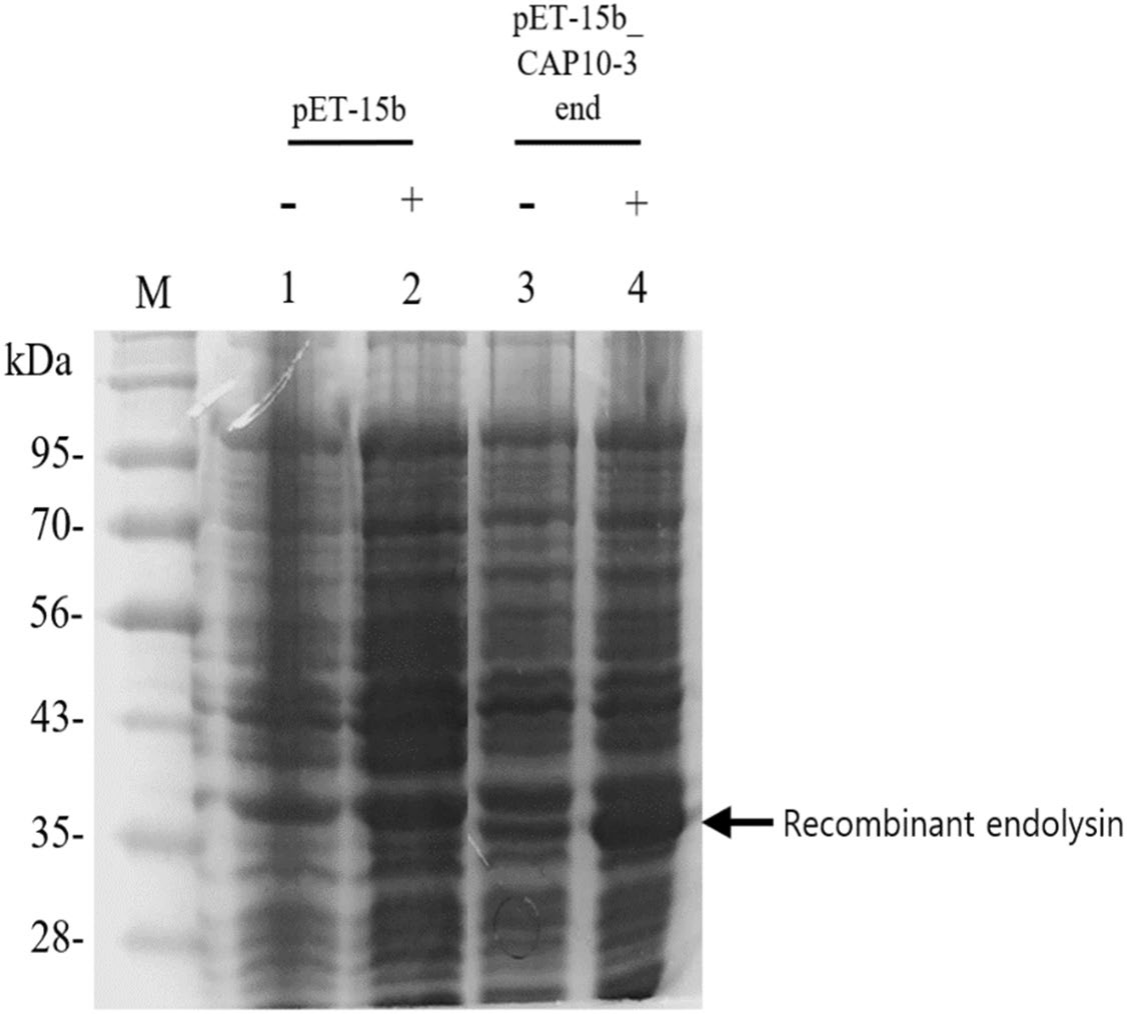
Production of a recombinant endolysin from *Escherichia coli* BL21(DE3) (pET-15b_CAP10-3 end) on an SDS-PAGE gel. M, prestained size marker (GangNam-Stain™, Intron biotechnology, Inc., Korea); Lane 1, 2 for *E. coli* BL21(DE3) (pET-15b); Lane 3, 4 for *E. coli* BL21(DE3) (pET-15b_CAP10-3 end). −, No addition of IPTG; +, Addition of IPTG. A full-size image of the SDS–PAGE gel is available in the Supplementary Information S1.

**Figure 3:**
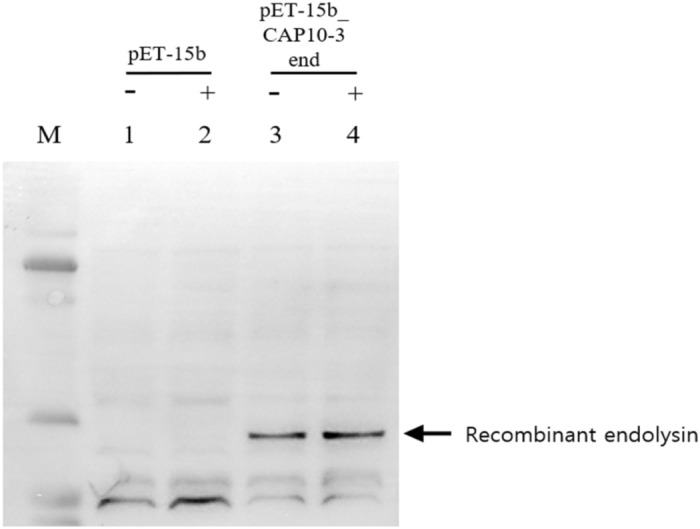
Western blot analysis for the recombinant endolysin from *Escherichia coli* BL21(DE3) (pET-15b_CAP10-3 end) by using an anti-His tag antibody. M, prestained size marker (GangNam-Stain™); Lane 1, 2 for *E. coli* BL21(DE3) (pET-15b); Lane 3, 4 for *E. coli* BL21(DE3) (pET-15b_CAP10-3 end). −, No addition of IPTG; +, addition of IPTG. A full-size image of the blot membrane is available in the Supplementary Information S1.

The recombinant endolysin gene was successfully expressed in *E. coli* BL21(DE3) (pET-15b_CAP 10-3 end) under 1 mM IPTG induction, which was confirmed on an SDS-PAGE gel where the expressed recombinant endolysin (MW, ~ 33 kDa) band was located near 35 kDa of size marker (GangNam-Stain™, Intron Biotechnology, Inc.). However the band was also detected from the cell pellet without IPTG induction, which means the pET-15b expression system used in this study was not properly controlling the gene expression (Fig. 2). To specifically detect the recombinant His-tagged endolysin, Western blot assay was performed and the specific band for the protein was detected on the transferred membrane. Like the SDS-PAGE result, the band was also detected from the cell lysate without IPTG induction (Fig. 3).

### Antibacterial activity of recombinant endolysin against *C. acnes*

**Figure 4:**
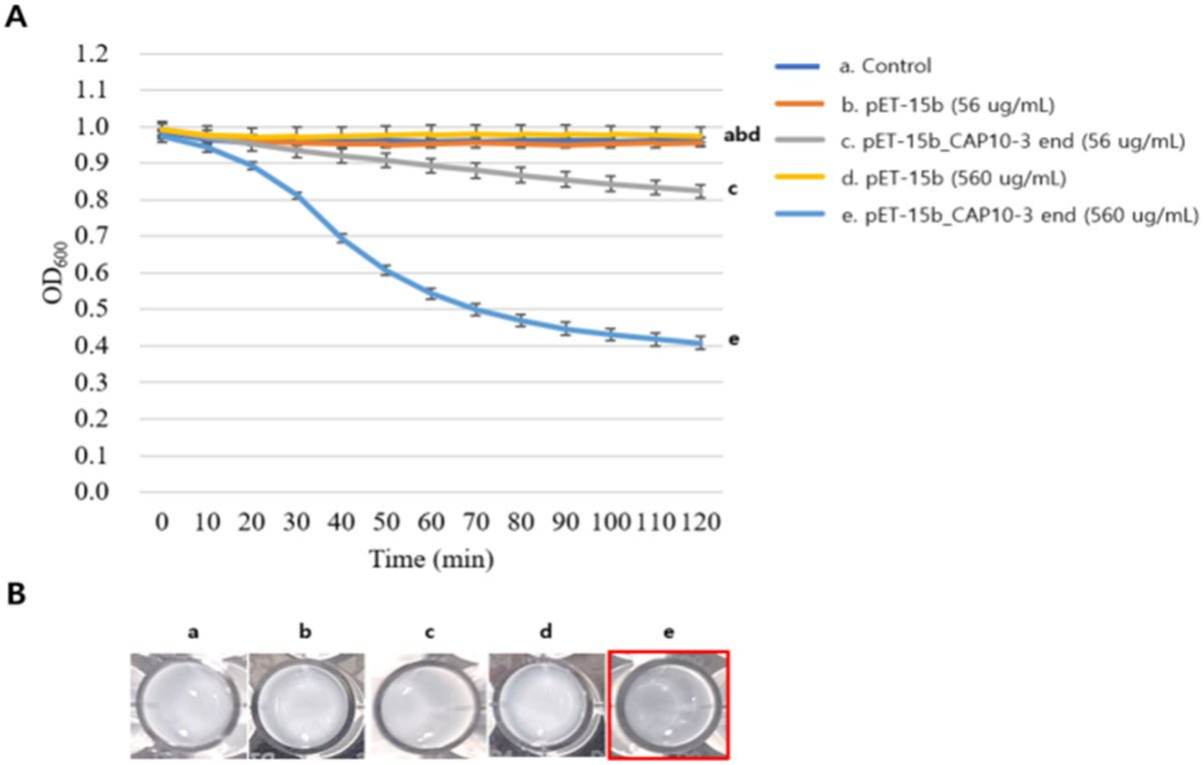
Change of optical density of *Cutibacterium acnes* KCTC 3314 culture by concentrated supernatants of cell lysates of recombinant *Escherichia coli* BL21(DE3) strains. (**A**) Optical densities during incubation time; (**B**) Transparency of cell culture in wells. Control, lysis buffer; pET-15b, *E. coli* BL21(DE3) (pET-15b); pET-15b_CAP10-3 end, *E. coli* BL21(DE3) (pET-15b_CAP10-3 end). The experiment was repeated 3 times and expressed as mean ± standard deviation.

**Figure 5:**
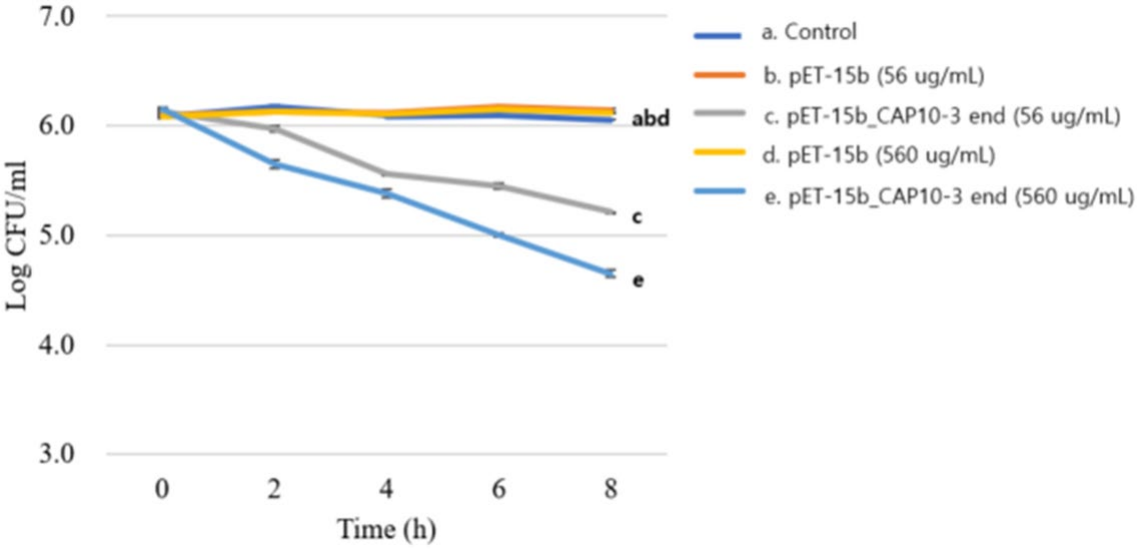
Change of viable cell count of *Cutibacterium acnes* KCTC 3314 culture by concentrated supernatants of cell lysates of recombinant *Escherichia coli* BL21(DE3) strains. Control, lysis butter; pET-15b, *E. coli* BL21(DE3) (pET-15b); pET-15b_CAP10-3 end, *E. coli* BL21(DE3) (pET-15b_CAP10-3 end). The experiment was repeated 3 times and expressed as mean ± standard deviation.

**Figure 6:**
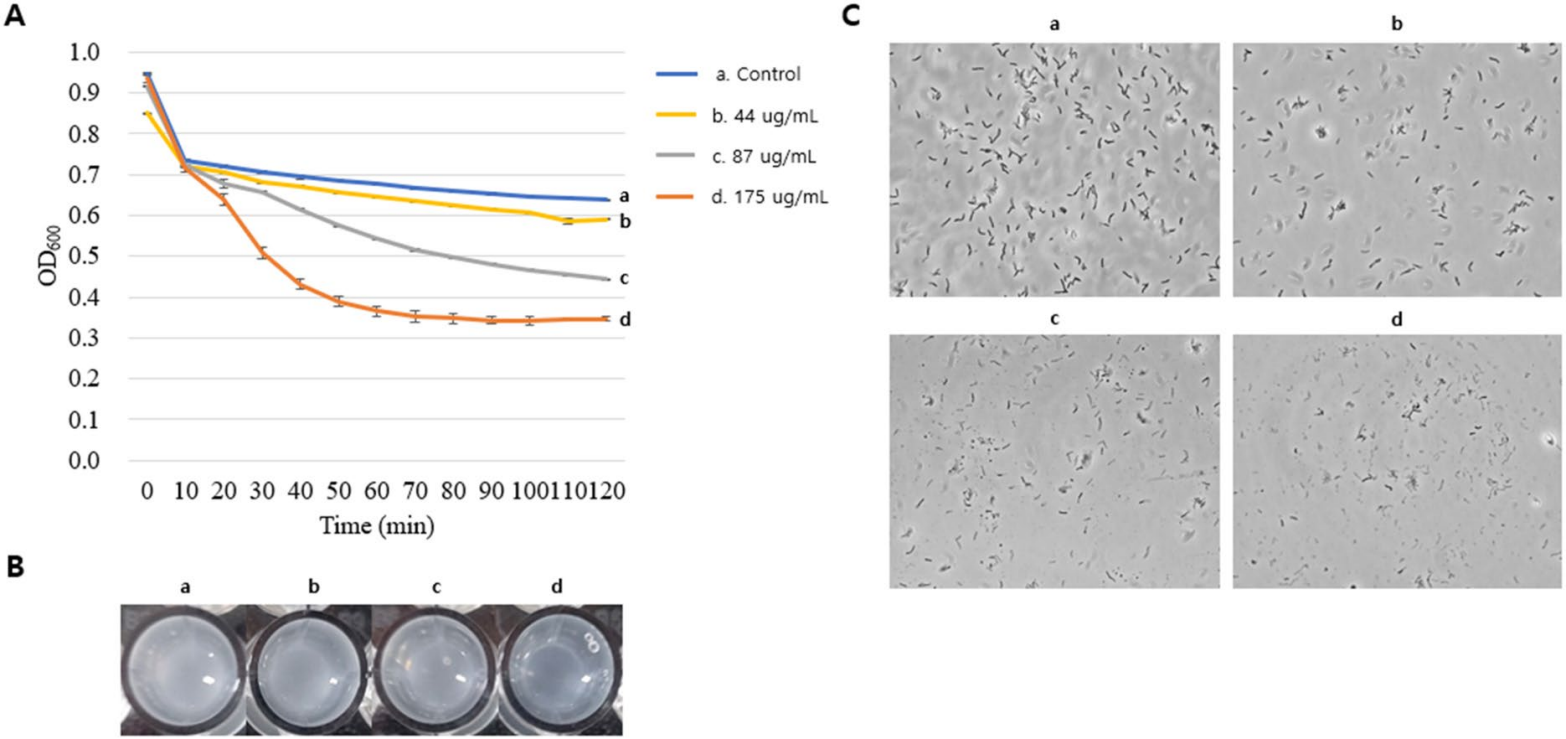
Change of optical density of *Cutibacterium acnes* KCTC 3314 culture by purified recombinant endolysin produced from *Escherichia coli* BL21(DE3) (pET-15b_CAP10-3 end). (**A**) Optical densities during incubation time; (**B**) Transparency of cell culture in wells; (**C**) Microscopic images. The experiment was repeated 3 times and expressed as mean ± standard deviation.

Antibacterial activity of recombinant endolysin against *C. acnes* was analyzed by using turbidity reduction method. For the beginning, the values among samples were not significantly different but at the middle phase (60 min) the optical densities started to decrease in the recombinant endolysin treatments with dose-dependent manner and at the final phase (120 min) the values were significantly different between treatment of concentrated supernatant of cell lysate for *E. coli* BL21(DE3) (pET-15b) and that of *E. coli* BL21(DE3) (pET-15b_CAP10-3 end). In particular, optical density value of treatment of concentrated supernatant of cell lysate for *E. coli* BL21(DE3) (pET-15b_CAP10-3 end) was decreased 0.97 to 0.41 and it was also reflected on culture clearance (Fig. 4). In the viable cell count test, the similar trend to optical density test was observed. Briefly, the viable cell count of treatment of concentrated supernatant of cell lysate for *E. coli* BL21(DE3) (pET-15b) was not significantly different when compared with the starting point during 8 h. However, the cell count in the treatment of concentrated supernatant of cell lysate for *E. coli* BL21(DE3) (pET-15b_CAP10-3 end) was dramatically decreased with a dose-dependent manner when compared with the starting point during 8 h. In particular, it started 6.15 log CFU/mL of *C. acnes* and decreased to 4.65 log CFU/mL in the treatment of 560 ug/mL of proteins from concentrated supernatant of cell lysate for *E. coli* BL21(DE3) (pET-15b_CAP10-3 end) with IPTG induction (Fig. 5). Based on the results, the recombinant endolysin has antimicrobial activity against *C. acnes*, which indicates the recombinant strategy is properly working as an active form. The purified recombinant endolysin which includes 6 × His in its N-terminal showed antimicrobial activity against *C. acnes* in the turbidity reduction test. Namely, the control group using the elution buffer reduced optical density at 600 nm of frozen *C. acnes* from 0.95 to 0.64, whereas 44, 87, 175 ug/mL of purified recombinant endolysin reduced it from 0.85 to 0.59, 0.92 to 0.45, and 0.94 to 0.35, respectively with a dose-dependent manner (Fig. 6). The culture clearance and microscope images where more cell debris were observed according to increase of concentration of the purified recombinant endolysin also supported the result (Fig. 6).

## Discussion

*C. acnes* is the major contributor in the pathogenesis of acne vulgaris. Increasing antibiotic resistance demands urgent need of exploring new treatment alternatives^8^. Phage therapy has the potential to be an effective and promising antibacterial option in humans as demonstrated by several studies^30–32^. In the current study, whole genome sequencing and characterization of *C. acnes*’ phage designated as CAP 10-3 bacteriophage which shows species specificity (Supplementary Table S1) were carried out. CAP 10-3 bacteriophage has a genome of 29.6 kb with a GC content of 53.86%. These findings agree with the previous reports which reported similar range of GC content and genome size of *C. acnes* phages^33–37^. CAP 10-3 shares a high degree of similarity (almost 90% nucleotide sequence) with *C. acnes* phage PAP 1-1. More than 70% of genes with confirmed functions showed a high homology of more than 85% with the CDSs of *C. acnes* phage PAP 1-1. Additionally, homology search using BLASTn showed that CAP 10-3 also shares a high degree similarity with at least 100 other *C. acnes* phages. Particularly, TMP (Tape measure protein used by phages to make channels for delivering their genetic material inside their host cell) showed the highest degree of similarity with the available *C. acnes* phage genomes. These findings are consistent with the previous report mentioning that genome of *C. acnes* phages showed more than 85% identity despite their collection from a wide range of geographic region over a 30 year time span^37^ and that TMPs are conserved in *Siphovirus* phages (which infect *C. acnes*)^3^. Limited genetic diversity, in fact, has been termed as an exception for phages infecting *C. acnes*^38^ since phages infecting other bacteria like *Pseudomonas*, *Staphylococcus*, *Acinetobacter*, *Mycobacterium* exhibit a wide range of diversity^39–42^.

One potential reason for the lack of diversity in these phages is low genetic diversity in their host strains as described by Tomida et al.^43^ which, in turn, is a result of particularly strict nature of the niche in which *C. acnes* is predominantly found i.e. pilosebaceous unit of human skin^44^.

Despite being specific endolysin have relatively broader range of activity as compared to their parent phage making them a promising antimicrobial agent^45^. Other concerns with phage therapy like possibility of mutation, immune response, the time and cost involved to commercialize their use can also be taken care of by using endolysins^46^. Although commercially available products utilizing endolysin as therapeutic agent are still uncommon, success of one such product *Staphefekt* and results of several preclinical studies which evaluated its safety and efficacy forecast a promising future for endolysin-based therapy^47–49^.

Analysis of antibacterial activity of recombinant endolysin showed that it successfully controlled *C. acnes* KCTC 3314 in a dose dependent manner i.e. higher the concentration of the protein greater was its lytic effect (Figs. 4, 5, 6). This observation has analogy to our recent study in which the phage PAP 1-1 also showed lytic activity against *C. acnes* in a similar dose dependent way^28^*.* Varotsou et al.^50^ have also recently reported dose dependent effect of recombinant endolysin originated from a *C. acnes* phage against several bacterial species. Our further literature research for use of recombinant endolysin against *C. acnes* failed to yield any other reports. Most of the available literature is centered on assessing the potential of endolysin against more notorious pathogens such as *Staphylococcus*, *Streptococcus*, *Pseudomonas* spp.^51^. Our study, therefore, is one of pioneering reports of using recombinant endolysin against *C. acnes.* Chandran et al.^52^ also reported a dose dependent effect of recombinant endolysin in which viable cell reduction of 1–3.5-log was reported by two different recombinant endolysins, which shows that different endolysins may vary in their lytic ability. Optical density at 600 nm in the turbidity reduction method are also comparable with the mentioned study^53^. Several other studies have also reported dose/concentration and time dependent cell lysis using endolysins, however with differing results against different bacterial species^45,54,55^ which suggests that different lysins may have various abilities of cell lysis depending on their intrinsic characteristics, target host and the medium in which these experiments are conducted.

In conclusion, the recombinant CAP 10-3 endolysin showed antibacterial acivity against *C. acnes* in a dose-dependent manner which indicates that it could be a promising therapeutic candidate to treat skin acne patient. However, further studies on improved solubility of the recombinant endolysin in *E. coli* strains, improved purification yields, using different bacterial hosts for production, etc. are still necessary for commercialization.

### Supplementary Information

Supplementary Information.

## Data Availability

The datasets generated and analyzed during the current study are available in the GenBank repository with accession no. OR039357. In particular CAP 10-3 endolysin amino acid sequence datum is available at the GenBank accession no. WIW76974.1.

## References

[1] Dessinioti C, Katsambas A (2017). *Propionibacterium acnes* and antimicrobial resistance in acne. Clin. Dermatol..

[2] Fitz-Gibbon S (2013). *Propionibacterium acnes* strain populations in the human skin microbiome associated with acne. J. Invest. Dermatol..

[3] Kim S, Song H, Jin JS, Lee WJ, Kim J (2022). Genomic and phenotypic characterization of *Cutibacterium acnes* bacteriophages isolated from acne patients. Antibiotics.

[4] Eishi Y (2023). Potential association of *Cutibacterium acnes* with sarcoidosis as an endogenous hypersensitivity infection. Microorganisms.

[5] Patel PM, Camps NS, Rivera CI, Gomez I, Tuda CD (2022). *Cutibacterium acnes*: An emerging pathogen in culture negative bacterial prosthetic valve infective endocarditis (IE). IDCases.

[6] Mayslich C, Grange PA, Dupin N (2021). *Cutibacterium acnes* as an opportunistic pathogen: An update of its virulence-associated factors. Microorganisms.

[7] Castillo DE, Nanda S, Keri JE (2019). *Propionibacterium (Cutibacterium) acnes* bacteriophage therapy in acne: Current evidence and future perspectives. Dermatol. Ther. (Heidelb).

[8] Kraft J, Freiman A (2011). Management of acne. Can. Med. Assoc. J..

[9] Ross, J. I. *et al.* Phenotypic and genotypic characterization of antibiotic-resistant *Propionibacterium acnes* isolated from acne patients attending dermatology clinics in Europe, the U.S.A., Japan and Australia. *Br. J. Dermatol.* **144**, 339–346. 10.1046/j.1365-2133.2001.03956.x (2001). 10.1046/j.1365-2133.2001.03956.x 11251569

[10] Eady EA, Gloor M, Leyden JJ (2003). *Propionibacterium acnes* resistance: A worldwide problem. Dermatology.

[11] Coates P (2002). Prevalence of antibiotic-resistant propionibacteria on the skin of acne patients: 10-year surveillance data and snapshot distribution study. Br. J. Dermatol..

[12] Tzellos T, Zampeli V, Makrantonaki E, Zouboulis CC (2011). Treating acne with antibiotic-resistant bacterial colonization. Expert Opin. Pharmacother..

[13] Brives C, Pourraz J (2020). Phage therapy as a potential solution in the fight against AMR: Obstacles and possible futures. Palgrave Commun..

[14] Haq IU, Chaudhry WN, Akhtar MN, Andleeb S, Qadri I (2012). Bacteriophages and their implications on future biotechnology: A review. Virol. J..

[15] Lin DM, Koskella B, Lin HC (2017). Phage therapy: An alternative to antibiotics in the age of multi-drug resistance. World J. Gastrointest. Pharmacol. Ther..

[16] Kutateladze M, Adamia R (2010). Bacteriophages as potential new therapeutics to replace or supplement antibiotics. Trends Biotechnol..

[17] Bourdin G (2014). Coverage of diarrhoea-associated *Escherichia coli* isolates from different origins with two types of phage cocktails. Microb. Biotechnol..

[18] Donlan RM (2009). Preventing biofilms of clinically relevant organisms using bacteriophage. Trends Microbiol..

[19] Yang H (2012). Existence of separate domains in lysin PlyG for recognizing *Bacillus anthracis* spores and vegetative cells. Antimicrob. Agents Chemother..

[20] Chan BK (2016). Phage selection restores antibiotic sensitivity in MDR *Pseudomonas aeruginosa*. Sci. Rep..

[21] Shen Y (2016). A bacteriophage endolysin that eliminates intracellular streptococci. Elife.

[22] Nelson D, Loomis L, Fischetti VA (2001). Prevention and elimination of upper respiratory colonization of mice by group A streptococci by using a bacteriophage lytic enzyme. Proc. Natl. Acad. Sci. USA.

[23] Loeffler JM, Djurkovic S, Fischetti VA (2003). Phage lytic enzyme Cpl-1 as a novel antimicrobial for pneumococcal bacteremia. Infect. Immun..

[24] Entenza JM, Loeffler JM, Grandgirard D, Fischetti VA, Moreillon P (2005). Therapeutic effects of bacteriophage Cpl-1 lysin against *Streptococcus pneumoniae* endocarditis in rats. Antimicrob. Agents Chemother..

[25] Cheng Q, Nelson D, Zhu S, Fischetti VA (2005). Removal of group B streptococci colonizing the vagina and oropharynx of mice with a bacteriophage lytic enzyme. Antimicrob. Agents Chemother..

[26] McCullers JA, Karlström Å, Iverson AR, Loeffler JM, Fischetti VA (2007). Novel strategy to prevent otitis media caused by colonizing *Streptococcus pneumoniae*. PLoS Pathog..

[27] Yoong P, Schuch R, Nelson D, Fischetti VA (2004). Identification of a broadly active phage lytic enzyme with lethal activity against antibiotic-resistant *Enterococcus faecalis* and *Enterococcus faecium*. J. Bacteriol..

[28] Han M-H, Khan SA, Moon G-S (2023). *Cutibacterium acnes* KCTC 3314 growth reduction with the combined use of bacteriophage PAP 1-1 and nisin. Antibiotics.

[29] Hecht, A. *et al.* Measurements of translation initiation from all 64 codons in *E. coli*. *Nucleic Acids Res.* **45**, 3615–3626. 10.1093/nar/gkx070 (2017). 10.1093/nar/gkx070 PMC5397182 28334756

[30] Górski A, Targońska M, Borysowski J, Weber-Dąbrowska B (2009). The potential of phage therapy in bacterial infections of the eye. Ophthalmologica.

[31] Rhoads, D. D. *et al.* Bacteriophage therapy of venous leg ulcers in humans: Results of a phase I safety trial. *J. Wound Care* **18**, 237–243. 10.12968/jowc.2009.18.6.42801 (2009). 10.12968/jowc.2009.18.6.42801 19661847

[32] Jun JW (2014). Bacteriophage therapy of a Vibrio parahaemolyticus infection caused by a multiple-antibiotic-resistant O3:K6 pandemic clinical strain. J. Infect. Dis..

[33] Brown TL, Petrovski S, Dyson ZA, Seviour R, Tucci J (2016). The formulation of bacteriophage in a semi solid preparation for control of *Propionibacterium acnes* growth. PLoS One.

[34] Farrar MD (2007). Genome sequence and analysis of a *Propionibacterium acnes* bacteriophage. J. Bacteriol..

[35] Lam HYP (2021). Therapeutic effect of a newly isolated lytic bacteriophage against multi-drug-resistant *Cutibacterium acnes* infection in mice. Int. J. Mol. Sci..

[36] Liu J (2015). The diversity and host interactions of *Propionibacterium acnes* bacteriophages on human skin. ISME J..

[37] Marinelli, L. J. *et al.**Propionibacterium acnes* bacteriophages display limited genetic diversity and broad killing activity against bacterial skin isolates. *mBio* **3**, 1 (2012). 10.1128/mBio.00279-12. 10.1128/mBio.00279-12 PMC3448167 23015740

[38] Cheng L (2018). Complete genomic sequences of *Propionibacterium freudenreichii* phages from Swiss cheese reveal greater diversity than *Cutibacterium* (formerly *Propionibacterium*) *acnes* phages. BMC Microbiol..

[39] Kwan T, Liu J, DuBow M, Gros P, Pelletier J (2006). Comparative genomic analysis of 18 *Pseudomonas aeruginosa* bacteriophages. J. Bacteriol..

[40] Kwan T, Liu J, Dubow M, Gros P, Pelletier J (2005). The complete genomes and proteomes of 27 *Staphylococcus aureus* bacteriophages. Proc. Natl. Acad. Sci. USA.

[41] Oliveira H (2022). Genomic diversity of bacteriophages infecting the genus *Acinetobacter*. Viruses.

[42] Pope WH (2015). Whole genome comparison of a large collection of mycobacteriophages reveals a continuum of phage genetic diversity. eLife.

[43] Tomida S, Nguyen L, Chiu BH, Liu J, Sodergren E, Weinstock GM, Li H (2013). Pan-genome and comparative genome analyses of *Propionibacterium acnes* reveal its genomic diversity in the healthy and diseased human skin microbiome. MBio.

[44] Bek-Thomsen M, Lomholt HB, Kilian M (2008). Acne is not associated with yet-uncultured bacteria. J. Clin. Microbiol..

[45] Mayer MJ, Gasson MJ, Narbad A (2012). Genomic sequence of bacteriophage ATCC 8074–B1 and activity of its endolysin and engineered variants against *Clostridium sporogenes*. Appl. Environ. Microbiol..

[46] Murray E, Draper LA, Ross RP, Hill C (2021). The advantages and challenges of using endolysins in a clinical setting. Viruses.

[47] Abdelkader K, Gerstmans H, Saafan A, Dishisha T, Briers Y (2019). The preclinical and clinical progress of bacteriophages and their lytic enzymes: The parts are easier than the whole. Viruses.

[48] Jun SY (2017). Pharmacokinetics and Tolerance of the Phage endolysin-based candidate drug SAL200 after a single intravenous administration among healthy volunteers. Antimicrob. Agents Chemother..

[49] Totté, J. E. E., van Doorn, M. B. & Pasmans, S. G. M. A. Successful treatment of chronic *Staphylococcus aureus*-related dermatoses with the topical Endolysin Staphefekt SA.100: A report of 3 cases. *Case Rep. Dermatol.* **9**, 19–25. 10.1159/000473872 (2017). 10.1159/000473872 PMC5465516 28611631

[50] Varotsou C, Premetis GE, Labrou NE (2023). Characterization and engineering studies of a new endolysin from the *Propionibacterium acnes* bacteriophage PAC1 for the development of a broad-spectrum artilysin with altered specificity. Int. J. Mol. Sci..

[51] Roach DR, Donovan DM (2015). Antimicrobial bacteriophage-derived proteins and therapeutic applications. Bacteriophage.

[52] Chandran C (2022). *Lactococcus lactis* secreting phage lysins as a potential antimicrobial against multi-drug resistant *Staphylococcus aureus*. Peer J..

[53] Lee C, Kim J, Son B, Ryu S (2021). Development of advanced chimeric endolysin to control multidrug-resistant *Staphylococcus aureus* through domain shuffling. ACS Infect. Dis..

[54] Yuan Y (2021). The endolysin of the *Acinetobacter baumannii* phage vB_AbaP_D2 shows broad antibacterial activity. Microb. Biotechnol..

[55] Bhagwat A, Zhang F, Collins CH, Dordick JS (2021). Influence of bacterial culture medium on peptidoglycan binding of cell wall lytic enzymes. J. Biotechnol..

