# Supplementary material for: Expression of a recombinant endolysin from bacteriophage CAP 10-3 with lytic activity against Cutibacterium acnes
Source: Sci Rep. 2023 Sep 30;13:16430. doi: 10.1038/s41598-023-43559-z (PMC10542754; doi:10.1038/s41598-023-43559-z)

Supplementary Information file

Fig. 2 original


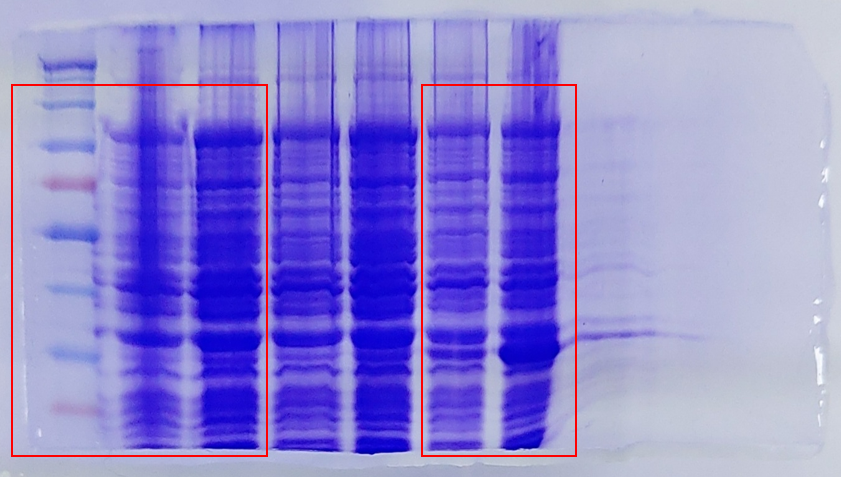


Fig. 3 original


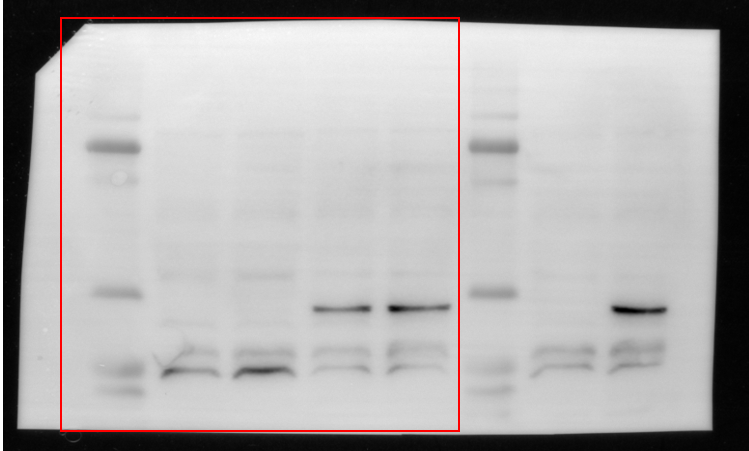


Supplemental Table S1. Antimicrobial spectrum of *Cutibacterium acnes* phage CAP 10-3


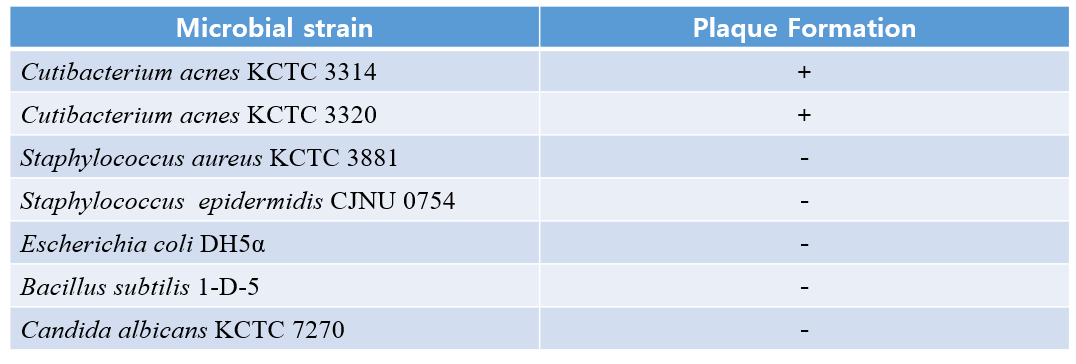

Supplement: Supplementary file 1 — Supplementary Information. [file 41598_2023_43559_MOESM1_ESM.docx]
